# Atomic-scale 3D imaging of individual dopant atoms in an oxide semiconductor

**DOI:** 10.1038/s41467-022-32189-0

**Published:** 2022-08-15

**Authors:** K. A. Hunnestad, C. Hatzoglou, Z. M. Khalid, P. E. Vullum, Z. Yan, E. Bourret, A. T. J. van Helvoort, S. M. Selbach, D. Meier

**Affiliations:** 1 Department of Materials Science and Engineering, NTNU Norwegian University of Science and Technology, 7491 Trondheim, Norway; 2 Department of Physics, NTNU Norwegian University of Science and Technology, 7491 Trondheim, Norway; 3 SINTEF Industry, 7034 Trondheim, Norway; 4 Department of Physics, ETH Zurich, Zürich, Switzerland; 5 Materials Sciences Division, Lawrence Berkeley National Laboratory, Berkeley, CA USA

**Keywords:** Semiconductors, Ferroelectrics and multiferroics, Ferroelectrics and multiferroics

## Abstract

The physical properties of semiconductors are controlled by chemical doping. In oxide semiconductors, small variations in the density of dopant atoms can completely change the local electric and magnetic responses caused by their strongly correlated electrons. In lightly doped systems, however, such variations are difficult to determine as quantitative 3D imaging of individual dopant atoms is a major challenge. We apply atom probe tomography to resolve the atomic sites that donors occupy in the small band gap semiconductor Er(Mn,Ti)O_3_ with a nominal Ti concentration of 0.04 at. %, map their 3D lattice positions, and quantify spatial variations. Our work enables atomic-level 3D studies of structure-property relations in lightly doped complex oxides, which is crucial to understand and control emergent dopant-driven quantum phenomena.

## Abstract (web summary)

Small variations in the density of dopants change the physical properties of complex oxides. Here, the authors resolve doping levels in three dimension, imaging the atomic sites that donors occupy in the small band gap semiconductor Er(Mn,Ti)O_3_.

## Introduction

The engineering of electronic responses with dopant atoms is essential for modern technology. The functionality of diodes and transistors, for example, relies on semiconductors where dopant atoms generate the free holes (p-type) or electrons (n-type) that define the transport properties. Despite their substantial impact on the conductivity, the number of dopant atoms is usually small, and even highly doped silicon contains just 1 dopant atom per ∼10^3^ Si atoms. In a more recent development, oxide-based semiconductors moved into focus as a particularly promising class of tunable systems for device applications^1^. Analogous to conventional semiconductors^2^, very low concentrations of dopant atoms can lead to pronounced changes in the electronic properties of oxide materials. The latter is reflected by doping-dependent studies on hexagonal manganites, where doping with aliovalent cations below 0.05 atomic percent (at.%) resulted in an order of magnitude lower electrical conductivity^3^. Furthermore, in complex oxides, strong correlations between charge, spin, and lattice degrees of freedom arise, promoting a wide variety of additional doping-induced effects, including insulator-metal transitions^4^, interfacial magnetism^5^, and superconductivity^6^.

In contrast to more than 70 years of research on conventional semiconductors, however, the incorporation of dopant atoms in complex oxides is much less explored. Importantly, because of the symmetry reduction and strong electronic correlations, individual dopants can do much more than only control the type and concentration of mobile charge carriers. For example, dopants can induce local strain and strain gradients, electrostatic fields, orbital reconstruction, and novel magnetic phases^7^. Furthermore, dopants may occupy different regular lattice or interstitial sites with drastically different consequences for the physical properties of the host material^8,9^. In order to master this level of complexity and understand emergent composition-driven phenomena and opportunities in oxide materials, a careful characterization of the dopant atoms is crucial. For this purpose, different experimental techniques, such as impedance spectroscopy^10^, Hall measurements^11^, and secondary ion mass spectrometry^12^ have been applied, sensing average doping levels down to parts per billion. Despite their high sensitivity, these measurements cannot be applied to probe small volumes, let alone the lattice position of individual dopants and their interactions at the local scale, as they lack the necessary spatial resolution. To image single dopant atoms within the lattice and quantify their concentration, scanning transmission electron microscopy (STEM) has been applied in combination with energy-dispersive X-ray spectroscopy (EDX)^13,14^. This correlated approach represents a breakthrough in the atomic-scale characterization of doped oxides, but it is limited to doping levels higher than a few at.%. Furthermore, it is inherently restricted to 2D projections along specific zone axes, prohibiting the full three-dimensional (3D) characterization of dopant atoms. Density functional theory (DFT) calculations are often applied to fill this gap and the progress in large-scale DFT modeling is continuously easing size limitations^15^ so that lower and lower doping levels can be calculated. The DFT calculations, however, are usually performed for the ground state structure without addressing effects that can arise during high-temperature crystal growth where both anions and cations are highly mobile and configurational entropy may favor, e.g., cation anti-sites and vacancies, dopant clustering and non-stoichiometry. This additional degree of complexity is not captured by DFT, reflecting the need for an experimental probe that can resolve the individual dopant atoms and, hence, clarify the atomic-scale structure.

Here, we overcome this fundamental limitation by utilizing the unique chemical accuracy and sensitivity of atom probe tomography (APT) to resolve the 3D lattice position of individual dopant atoms in the lightly doped narrow bandgap semiconductor Er(Mn,Ti)O_3_ (*E*_g_ ≈1.6 eV^16^). The model system has a nominal Ti concentration of 0.04 at.%, which falls into a regime that is no longer accessible with STEM-based imaging techniques and is about three orders of magnitude lower compared to previous APT work performed on highly doped classical semiconductors^17,18^. By performing APT experiments on multiple needle-shaped specimens, we quantify the actual local doping level and spatial distribution. Our data reveal substantial deviations of up to ≈50% from the nominal dopant concentration as defined by the chemical stoichiometry during synthesis, establishing APT as a powerful tool for quantitative imaging of otherwise elusive dopant levels in semiconducting oxides and semiconductors in general.

APT is widely applied in metallurgy, where it is used for element-specific 3D imaging^19^. A key development that allowed for expanding APT studies towards a wider range of materials, including poorly conducting and even insulating systems, was the advent of laser-assisted field evaporation. Intriguing recent examples are APT experiments performed on frozen water^20^, human enamel^21^, multivariate metal-organic frameworks^22^, and its application in geosciences^23^. Pioneering APT investigations on complex oxides were performed on Pb(Zr,Ti)O_3_ ceramics, NiFe_2_O_4_-LaFeO_3_ nanocomposites^24^, and AlGaO_3_ superlattices^25^ studying chemical composition, phase segregation, and site-preferences of impurity atoms, respectively. Furthermore, it is now established that atomic resolution can be achieved in complex oxides^26^, opening the door for high-resolution 3D imaging of individual dopant atoms in oxide semiconductors.

## Results

### Quantifying the dopant concentration in Er(Mn,Ti)O_3_

**Fig. 1:**
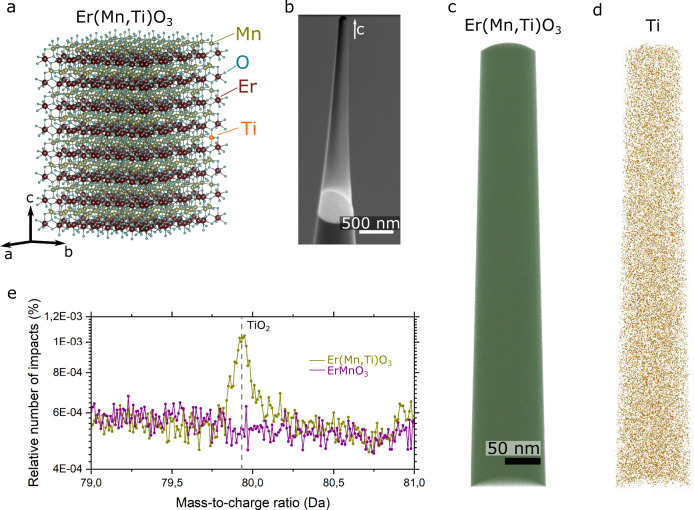
SEM and APT images of a needle-shaped Er(Mn,Ti)O_3_ sample. **a** Illustration of the crystallographic structure of Er(Mn,Ti)O_3_, showing the ratio between ErMnO_3_ lattice atoms and Ti dopants. For a dopant concentration of about 0.04 at.%, there is approximately one Ti atoms per 64 unit cells of ErMnO_3_. **b** Scanning electron microscopy (SEM) image of a FIB-cut needle specimen with the crystallographic *c*-axis oriented along the needle axis. **c** APT dataset gained from the needle shown in **b**, presenting all primary ionic elements (O – blue, Mn – yellow, Er – red, Ti – orange). **d** Subset of the APT dataset in **c**, displaying only the dopants, i.e., TiO_2_^+^ ionic species. **e** Comparison of a selected section from the APT mass spectra recorded on Er(Mn,Ti)O_3_ (yellow) and ErMnO_3_ (purple). The Er(Mn,Ti)O_3_ data shows a peak at ≈79.9 Da, which is characteristic for the TiO_2_^+^ species as indicated by the comparison with the spectrum of the undoped sample.

Our model system Er(Mn,Ti)O_3_ has a nominal Ti concentration of 0.04 at.% as illustrated in Fig. 1a. The material belongs to the family of hexagonal manganites, *R*MnO_3_ (*R* = Sc, Y, In, or Dy to Lu), and exhibits a layered structure of Er atoms and corner shared MnO_5_ bipyramids with *P*6_3_*cm* space group symmetry. The hexagonal manganites have been studied intensively with respect to their electric order^27^, magnetism^28^, and multiferroicity^29^, and their basic physical properties are well understood, which makes them ideal for reliably testing the 3D imaging of highly dilute doping levels. Enabled by the hexagonal crystal structure, the *R*MnO_3_ family offers outstanding chemical flexibility and allows for doping to induce p- or n-type semiconducting properties at will. This flexibility was utilized in previous studies, controlling the density and type of the majority charge carriers via aliovalent cation substitution on both the A- and B-sites, but without quantifying the applied doping levels and possible spatial variations^3,30,31^. The reason for this is the general difficulty to image individual acceptor or donor atoms with a concentration **≪**1 at.%. However, resolving such low doping levels is important when working with complex oxides, because dopant clustering, cation anti-sites, and non-stoichiometry may arise during high-temperature growth^32^, causing non-uniform electric and magnetic responses.

In order to access the dopant atoms in our Er(Mn,Ti)O_3_ sample and gain spatially resolved data in 3D, we analyze the chemical atomic-scale structure using APT. For this purpose, multiple needle-shaped specimens with a typical length of a few micrometers and a tip radius of less than 100 nm are extracted from an Er(Mn,Ti)O_3_ single crystal using a FIB. Needle-shaped specimens are the standard in APT to produce the high electric fields required for field evaporation of surface atoms (see Methods for details of the extraction procedure). A representative SEM image of one of the FIB-cut needles used in our APT experiments is displayed in Fig. 1b.

All extracted needles are oriented along the crystallographic *c*-axis as we confirm by electron diffraction (see Supplementary Fig. 1). We begin the discussion of the APT results with the 3D distribution of the different atomic species in the analyzed volume. To reduce atomic motion and achieve optimal spatial resolution, all APT measurements are performed at cryogenic temperature (see Methods for details). Figure 1c shows the APT reconstruction including all atoms, whereas only the Ti dopants are presented in Fig. 1d. The data suggests a homogeneous Ti distribution, which we confirm using radial distribution functions (RDF, Supplementary Fig. 2) and first nearest-neighbor analysis (1NN, Supplementary Fig. 3). The mass-to-charge ratio of the ions recorded during the APT field evaporation process is displayed in Fig. 1e along with data recorded for undoped ErMnO_3_. The mass spectra are centered around ≈79.9 Da, where a peak is observed only for Er(Mn,Ti)O_3_. This peak is characteristic of the TiO_2_^+^ ionic species (see Supplementary Fig. 4 for the complete mass spectra and Supplementary Fig. 5 for the secondary Ti isotopes) and, combined with the TiO^+^ ionic species, corresponds to a Ti concentration of 0.0086 at.%. In addition, Ti ions contribute to the TiO^2+^ peak, which partly overlaps with the O_2_^+^ peak in the mass spectrum (Supplementary Fig. 6). Accounting also for the ions associated with this Ti-related peak, we find a total Ti concentration of 0.0224 at.%, which translates into an average distance between Ti atoms of 2.08 nm for the investigated volume (Supplementary Fig. 3). To confirm that this concentration value is representative for our Er(Mn,Ti)O_3_ crystal, we measure multiple APT needles from different locations as presented in Supplementary Fig. 7, revealing a Ti concentration of 0.0239 ± 0.0045 at.%, which is about 50% lower than the nominal doping level as defined by the chemical stoichiometry during synthesis. In summary, the results in Fig. 1d (and Supplementary Figs. 2, 5) demonstrate a homogeneous Ti distribution, excluding clustering effects, chemical gradients, and other chemical inhomogeneities that may obscure the local electronic properties. However, we find that the Ti concentration is consistently lower than the nominal one, deviating by ≈50% for the investigated region. In fact, deviations from the nominal doping level are, in general, expected when crystals are derived from stoichiometric powders but are usually not detected due to the insensitivity of most characterization techniques to subtle compositional variations^33^. The results thus underline the need for careful quantitative investigations when it comes to lightly doped complex oxides and doping-driven phenomena in this class of materials.

### Atomically resolved APT measurements of the Er, Mn, and O lattice planes

**Fig. 2:**
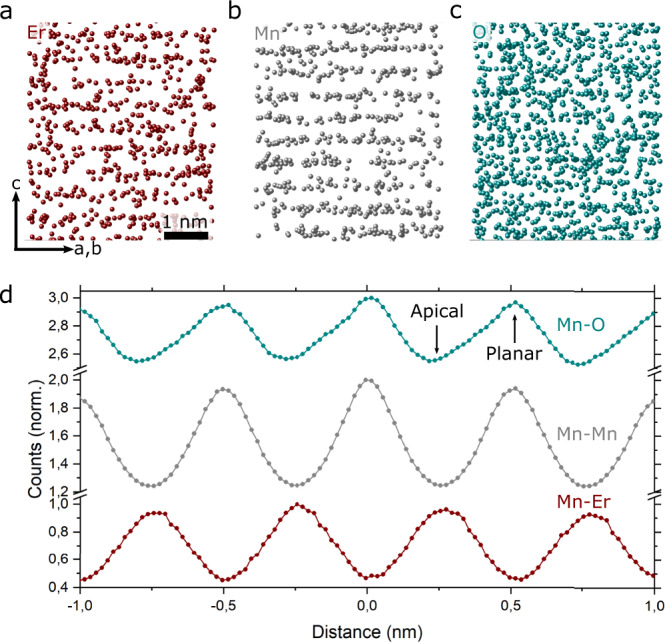
3D imaging of the Er, Mn, and O lattice atoms in Er(Mn,Ti)O_3_. **a** APT reconstruction showing the Er lattice planes (Er^3+^, Er^2+^, ErO^2+^, and ErO^+^) from a region in the pole (see Supplementary Fig. 8). The volume is oriented so that the layered structure of Er atoms along the crystallographic *c*-axis is visible, consistent with TEM data (Supplementary Fig. 9). **b**, **c** Same as in **a** for Mn (Mn^2+^ and Mn^+^) and O (O^+^) atoms, respectively. **d** SDMs of the lattice atoms, showing the distance between Mn and O (Mn-O) and Mn and Er (Mn-Er) with Mn serving as reference (Mn-Mn).

Next, we consider the site-specific distribution of the different atomic species at the local scale, beginning with the ErMnO_3_ lattice atoms (Fig. 2). In the *c*-axis oriented needles, atomic planes of Er and Mn are readily resolved in the APT experiment as presented in Fig. 2a and 2b; Fig. 2c displays the O atoms. To determine the atomic positions, we calculate spatial distribution maps (SDMs) in the evaporation direction *c*-axis as shown in Fig. 2d (see Methods for details). The SDMs are calculated for a volume of about 10 nm × 10 nm × 10 nm, corresponding to about 60,000 ions, and reveal the distance of the Er and O atomic planes relative to the Mn atoms measured along the crystallographic *c*-axis (Δz || *c*). The Mn planes serve as a reference system so that Δz(Mn-Mn) = 0. We observe maxima in the counts for Δz(Er-Mn) at about ±0.25 nm with a periodicity of 0.51 nm, indicating that the Er planes are located between the Mn planes, consistent with the high-angle annular dark-field scanning transmission electron microscopy (HAADF-STEM) image in Supplementary Fig. 9. Furthermore, the SDMs show that single O atoms (16 Da ionic species) are predominantly observed from the planar O positions, i.e., Δz(O-Mn) = 0, whereas apical O atoms are usually evaporated as ErO_x_ species (Supplementary Fig. 4). In conclusion, Fig. 2 shows that the 3D positions of the ErMnO_3_ lattice atoms are readily resolved in the APT experiment, enabling a detailed analysis of the incorporation of the Ti dopants.

### 3D imaging of solute Ti dopant atoms

**Fig. 3:**
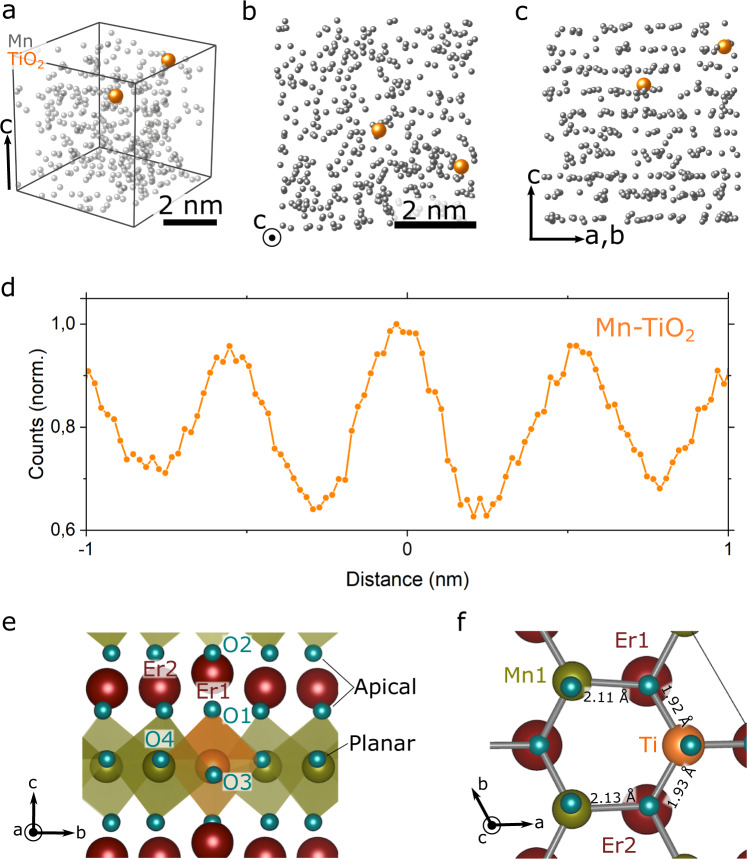
3D Imaging of individual Ti dopant atoms in Er(Mn,Ti)O_3_. **a** APT reconstruction from a region in the pole presenting the Mn (semi-transparent gray) atoms and Ti (orange) atoms (TiO_2_^+^ species). **b** Same volume as shown in **a** viewed along the *c*-axis and **c** perpendicular to the *c*-axis so that the layered structure of the Mn atoms is visible. The data indicate that the Ti atoms are located within the Mn layers. **d** Spatial distribution maps (SDMs) reveal the distance between the Ti ions (TiO_2_^+^ species) and the Mn ions (Mn^+^ and Mn^2+^) in orange, with the latter serving as reference systems. **e**, **f** Calculated local structure around the Ti dopant, situated on the B-site of the hexagonal ErMnO_3_ system and viewed along the *a***-** and *c*-axis, respectively.

Following the same approach as for the ErMnO_3_ lattice atoms, the position of individual Ti dopants is determined as presented in Fig. 3, showing data from the same needle as evaluated in Fig. 2. For better visualization, Fig. 3a displays a representative volume fraction of the much larger dataset gained from the [001]-pole; Fig. 3b, c show corresponding projections along and perpendicular to the crystallographic *c*-axis, respectively. Here, in addition to the Mn atoms (gray), the Ti atoms (or TiO_2_ ionic species) are shown in orange. Importantly, the spatially resolved APT data indicates that the Ti dopants have a propensity to sit within the Mn lattice planes as predicted by zero-Kelvin density functional theory (DFT) calculations^3^. To corroborate this visual impression and gain statistically significant information on the lattice position of the Ti atoms, we calculate SDMs over the entire needle length (>600 nm) and use more than 300 TiO_2_ ions to determine the site preference as shown in Fig. 3d. Furthermore, we repeat the experiment with multiple needles extracted from different positions (Supplementary Fig. 10). Using the Mn layers as a reference, we find a peak for Δz(TiO_2_-Mn) = 0 in all SDMs, which confirms that the Ti atoms are located in the Mn lattice planes. Figure 3 and Supplementary Fig. 10 thus provide direct experimental evidence that the Ti dopants are replacing Mn atoms. We note that we restrict the analysis to TiO_2_^+^ as this peak in the mass spectrum gives the best signal-to-noise ratio and, in contrast to the TiO^2+^ species, does not show substantial overlap with other peaks. As a consequence, and because the evaporation of Ti produces the same single or molecular ions independent of the host material and its lattice position^34–37^, this restriction improves the reliability of the SDMs without restricting the generality of our conclusions (see also Supplementary Fig. 11).

Complementary DFT calculations on a 540 atom 3 × 3 × 2 supercell of ErMnO_3_ with 1 of 108 Mn atoms substituted with Ti (≈0.19 at.%) are shown in Fig. 3e,f. This model approaches a doping level comparable with the experimentally studied system and provides additional insight into the local structure around the dopant atoms. Substantial atomic displacements are only found for planar oxygen (O3 and O4 in Fig. 3f) in the first coordination shell of the Ti dopant (Fig. 3e). Compared to the undoped structure, the Ti-O bond length is shorter, whereas Mn-O bonds (Fig. 3f) are subtly elongated, indicating a partial reduction of adjacent Mn caused by Ti^4+^ as a donor dopant in the Mn^3+^ sublattice. The strain field associated with the Ti atoms quickly decays (Supplementary Fig. 12), which explains why they are very difficult to observe in structural microscopy measurements that lack the chemical sensitivity of APT.

## Discussion

Our APT-based study of highly dilute individual dopant atoms can readily be transferred to other complex oxides and atomic species, expanding investigations of oxide semiconductors into the realm of atomic-scale 3D imaging. The chemical sensitivity of APT enables quantitative 3D imaging of otherwise elusive doping levels in chemically complex host materials. The latter is particularly important for oxides as they are usually synthesized at elevated temperatures, where configurational entropy promotes the formation of lattice defects. In addition, thermal gradients and cooling rates can play a crucial role in the concentration and distribution of dopant atoms, which makes it difficult to adequately model them, requiring atomically resolved measurements in combination with high chemical accuracy and sensitivity. Intriguing application opportunities also exist for doped oxide thin films and heterostructures^38^, where APT has the potential to advance the study of doping-related inhomogeneities, gradient effects, as well as interface phenomena, including interdiffusion and accumulation/depletion of acceptor and donor atoms. The characterization of associated electronic properties becomes possible by performing, e.g., advanced scanning probe microscopy measurements on the APT needles prior to the tomography experiment. The general feasibility of this strategy is demonstrated by recent atomic force microscopy experiments^39^, which can readily be customized to access the local transport properties, electrostatics, and electromechanical responses. Aside from the imaging of highly dilute dopant atoms in semiconductors, the APT approach can be applied to topological insulators and superconductors to understand the impact of individual dopant atoms^8,9,31^. Furthermore, it may shed light on doping-related magnetic phenomena, such as the Kondo effect^40^ and order-disorder transitions in multiferroics^41^, adding an additional dimension to the atomic-scale investigation of emergent phenomena in complex oxides.

## Methods

### Sample preparation and characterization

High-quality ErMnO_3_ and ErMn_1-x_Ti_x_O_3_ (x = 0.002) single crystals were grown by the pressurized floating-zone method^32^, oriented by Laue diffraction, and then cut to achieve oriented surfaces with the surface normal, *n*, parallel to the crystallographic *c*-axis. Samples were lapped and polished using silica slurry (particle size 20 nm) to obtain flat surfaces with sub-nanometer surface roughness. From the polished surfaces, APT needles with a tip radius ≲100 nm were prepared at 30 kV (and final polishing at 2 kV) using a Thermo Fisher Scientific G4 DualBeam UX Focused Ion Beam (FIB) analogous to the procedure described in ref. 42. All APT needles were prepared with the *c*-axis along the needle axis as this gives the optimal spatial resolution due to the layer-by-layer evaporation, in contrast to the lateral resolution affected by trajectory aberrations^19^. Cross-section TEM samples were also prepared using the same FIB, and EDX was done to confirm the presence of Ti (Supplementary Fig. 9). Carbon was used as a protection layer on the regions of interest. The first part of the protection layer was deposited with electron beam-assisted deposition to avoid Ga^+^ implantation and beam damage to the top of the oxide. All coarse thinning was performed at 30 kV acceleration voltage for the ion beam. Final thinning was first done at 5 kV and then at 2 kV on either side of the lamellae to minimize the surface damaged layer.

### Transmission electron microscopy and energy-dispersive X-ray spectroscopy

To analyze the sample quality and exclude, e.g., amorphization and structural damage from the FIB, selected specimens were inspected with TEM (Supplementary Fig. 1) using a JEOL 2100 F Field Emission Gun (FEG) microscope, operating at 200 kV. High-angle annular dark-field scanning transmission electron microscopy (HAADF-STEM) imaging of the lattice was performed on a double-Cs aberration corrected cold FEG JEOL ARM200FC at 200 kV. Images were acquired with beam semi-convergence angles of 27.4 mrad and a beam current of 21 pA. The inner and outer semi-collection angles were 51 and 203 mrad, respectively. Energy-dispersive X-ray spectroscopy (EDX) was performed with a 100 mm^2^ Centurio detector, covering a solid angle of 0.98 sr. EDX line scans were performed with a 110 pA beam current and 1.0 s dwell time for each pixel. No visible beam damage could be observed after the EDX line scans. The EDX data were analyzed with DigitalMicrograph, version 2.32.

### APT data collection

For the APT measurements, a Cameca LEAP 5000XS was used, operating in laser mode. All measurements were performed at cryogenic temperature, i.e., a temperature of 25–50 K. The field evaporation process was triggered using femtosecond laser pulses. By applying such laser pulses, the apex of the APT needle temporarily heats up, as discussed, e.g., in ref. 43. Following this established procedure, ions were controllably evaporated and the time-of-flight was measured. Spatial positions were recorded with a 2D detector and determined by the electrostatic field lines of the specimen. The raw data consists of information on both time-of-flight, directly linked to the charge-to-mass ratio, and the detector impact position related to the original position on the tip prior to evaporation. The histogram (i.e., mass spectrum) and detector events for a representative APT measurement are shown in Supplementary Fig. 4 and Supplementary Fig. 8, respectively. Laser pulses with a frequency of 250 kHz and energy between 2 and 30 pJ were used. The detection rate was set between 0.5 and 2%, meaning that, on average, 5–20 atoms were detected every 1000 pulse.

### APT data reconstruction and analysis

For the reconstruction of raw APT data from the different needles into 3D datasets, the software Cameca IVAS 3.6.12 was used. Reconstruction was done in voltage mode with an image compression factor of ≈1.8, a field reduction factor of ≈2.8, and an evaporation field of Mn (30 V/nm). The parameters were fine-tuned using spatial distribution map analysis so that accurate distances of atomic planes are measured in the reconstructed volume. For the peak at 16 Da, which could correspond to either O^+^ or O_2_^2+^, this is ranged as O^+^ and not O_2_^2+^ following the discussion in refs. 44,45.

### Spatial distribution maps (SDMs)

For the different needles compared in this work, SDMs are calculated by iterating through all the atoms, calculating the distance between atoms of one selected species and a reference species along a specific analysis direction. The distances are summed to generate a histogram, as shown in Fig. 2d^46^. To optimize the single-to-noise ratio, the analysis direction was chosen to be perpendicular to the atomic planes. The Mn atoms (27.5 Da) are used as references for all SDMs. For the matrix SDM, smaller volumes of about 10 nm × 10 nm × 10 nm were used, whereas for the dopants, much larger volumes of 300 nm × 10nm × 10 nm from the [100] pole region were considered (Supplementary Fig. 8). Only the major single ionic species were used for the matrix, i.e., O from 16 Da (1+ species), Mn from 27.5 Da (2+ species), and Er from 55 Da (3+ species). The Norwegian Atom Probe App (NAPA) software was used for all SDM analysis. NAPA was developed by C. H. and is an open access software for APT data treatment (https://www.ntnu.edu/ima/research/apt).

### Density functional theory (DFT) calculations

Our previous DFT calculations using 2 × 2 × 1 supercells confirmed that Ti in ErMnO_3_ occupies the Mn site, acting as a donor and reducing the bulk p-type conductivity^3^. Here, we used 540 atom 3 × 3 × 2 supercells with a more realistic Ti dopant concentration of x = 1/108 in ErMn_1-x_Ti_x_O_3_. The calculations were done using VASP^47,48^ with the projector augmented wave (PAW)^49^ method and the PBEsol functional^50^. For Er, Mn, Ti, and O, 9, 13, 12, and 6 electrons, respectively, were treated as valence electrons. Gamma point calculations were performed with a plane-wave cutoff energy of 550 eV and the geometry was relaxed until the forces on each ion were less than 0.03 eV/Å. A Hubbard *U* of 5 eV was applied to Mn 3*d* states to reproduce the experimental bandgap and lattice parameters^3^, and collinear frustrated antiferromagnetic order^51^ was imposed on the Mn sublattice.

### Reporting summary

Further information on research design is available in the Nature Research Reporting Summary linked to this article.

## Supplementary information

Supplementary Information

Reporting Summary

Lasing Reporting Summary

## Data Availability

All data generated or analyzed during this study are included in this published article and its Supplementary Information files. Further information is also available from the corresponding authors upon reasonable request.
