# Supplementary material for: Atomic-scale 3D imaging of individual dopant atoms in an oxide semiconductor
Source: Nat Commun. 2022 Aug 15;13:4783. doi: 10.1038/s41467-022-32189-0 (PMC9378652; doi:10.1038/s41467-022-32189-0)
Supplement: Supplementary file 1 — Supplementary Information [file 41467_2022_32189_MOESM1_ESM.pdf]

Supplementary material

Atomic-scale 3D imaging of individual dopant atoms in an oxide semiconductor

K. A. Hunnestad[1], C. Hatzoglou[1], Z. M. Khalid[1], P. E. Vullum[2,3], Z. Yan[4,5], E. Bourret[5], A. T. J. van Helvoort[2], S. M. Selbach[1] and D. Meier[1*]

[1]Department of Materials Science and Engineering, Norwegian University of Science and Technology (NTNU), 7491 Trondheim, Norway

[2] Department of Physics, Norwegian University of Science and Technology (NTNU), 7491 Trondheim, Norway

[3]SINTEF Industry, 7034 Trondheim, Norway

[4]Department of Physics, ETH Zurich, Zürich, Switzerland

[5]Materials Sciences Division, Lawrence Berkeley National Laboratory, Berkeley, CA, USA

*Corresponding author. Email: dennis.meier@ntnu.no

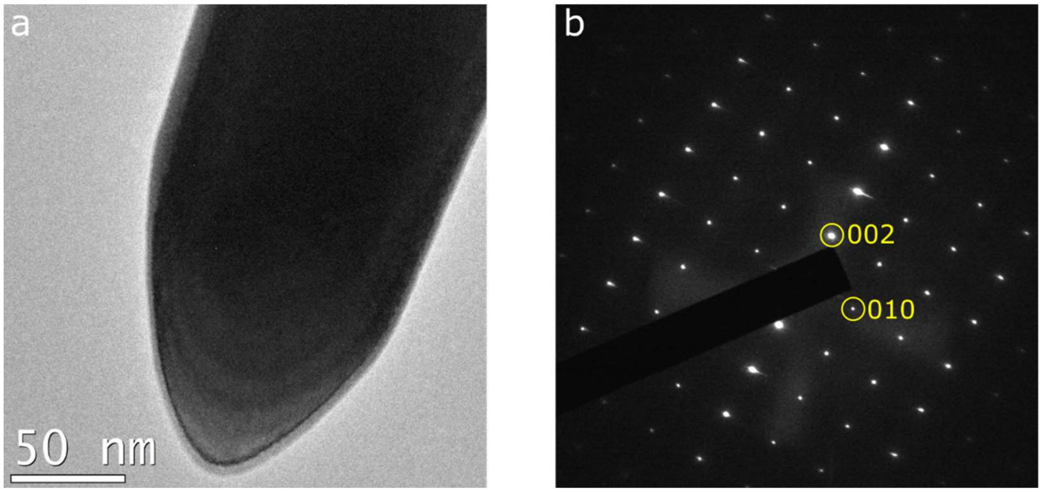

Supplementary Fig. 1 | Sample inspection with TEM. a, TEM image of the tip of a representative finished needle of $ErMnO_3$. The needle is slightly asymmetric, but no structural defects are observed and the bulk of the needle is crystalline. b, Selected-area electron diffraction (SAED) pattern of the needle in a. Only peaks associated to the hexagonal phase are seen, with no indication on secondary phases, reflecting the high-quality of the APT needle.

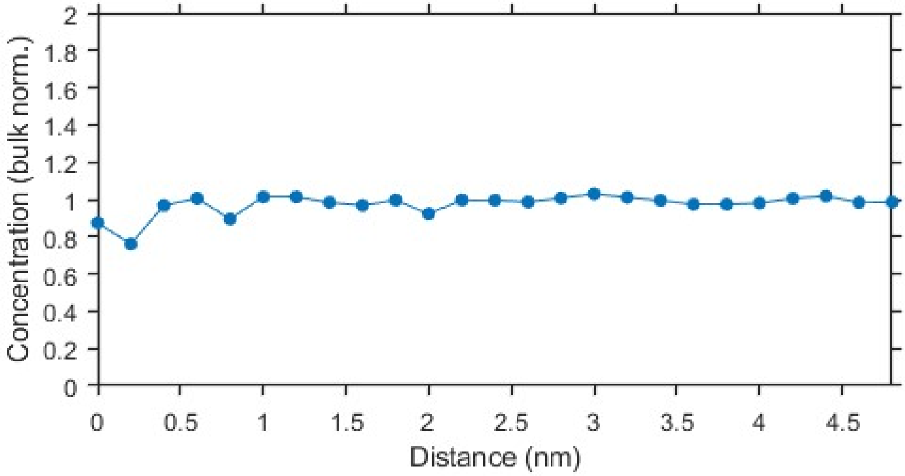

Supplementary Fig. 2 | Radial distribution function (RDF) analysis of Ti dopants. The graph displays the RDF analysis for Ti dopants for the sample presented in Fig. 1 in the main text. The calculation shows the chemical composition of Ti inside shells of increasing radii centered around the TiO_2 ions, normalized to the overall bulk composition, and displayed as a histogram. We note that only the TiO_2^+ ionic species was used as this was the most pronounced Ti species with the least amount of noise and background. The RDF analysis shows no substantial deviation from 1 at small distances, indicating a homogeneous distribution of Ti atoms.

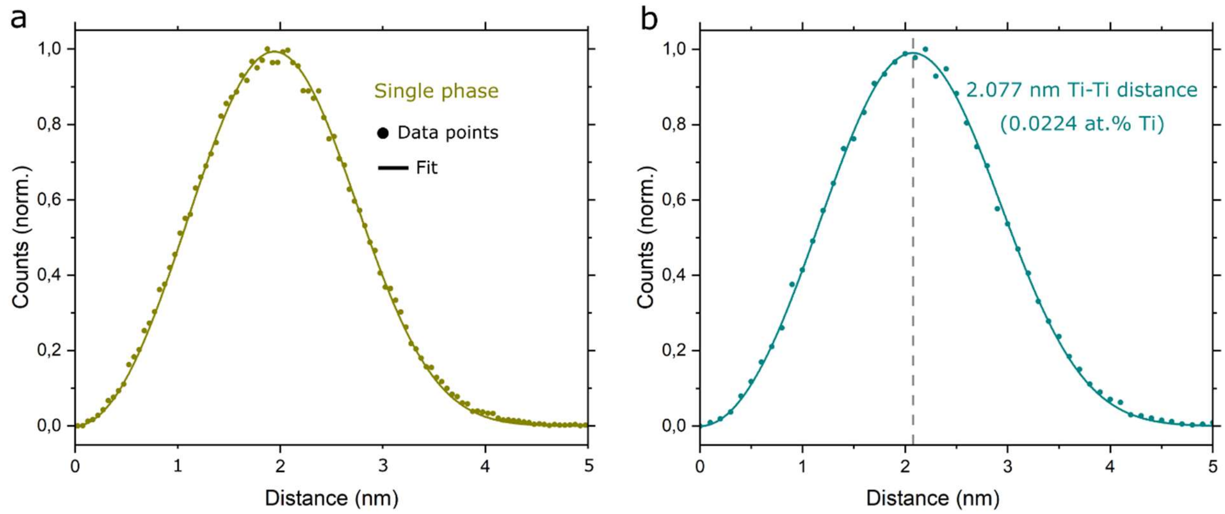

Supplementary Fig. 3 | First nearest neighbor (1NN) calculations for Ti dopants. a, 1NN calculations for the TiO and TiO_2 species based on the experimental data for the specimen shown in Fig. 1 of the main text. The solid line is a fit to the APT data, based on the model for a single-phase and homogenous distribution in Ref. [1], which was used to study secondary phases or clustering. The x-axis gives the separation distance between neighboring ions, whereas the y-axis shows the number of ions with this separation distance normalized to the maximum value. It is important to note that the experimental curve cannot be used to extract quantitative information as detection efficiency and background signals are not taken into account. The excellent agreement between model and experiment, however, allows to exclude clustering of the Ti atoms and Ti-related secondary phases. The latter implies that the 1NN distance of the Ti dopants can be calculated from the measured concentration, i.e., 0.0224 at. % for the sample in Fig. 1 of the main text as shown in b, corresponding to an average 1NN distance of 2.077 nm (solid line: fit to the data, same model as above). The deviation in 1NN distance between the experimental a and simulated b data is due to a combination of a relatively high background level

and a limited detection efficiency (Supplementary Fig. 13), in addition to a different selection of ionic species ((A) uses only the TiO_2^+ species, while (B) is based on all species).

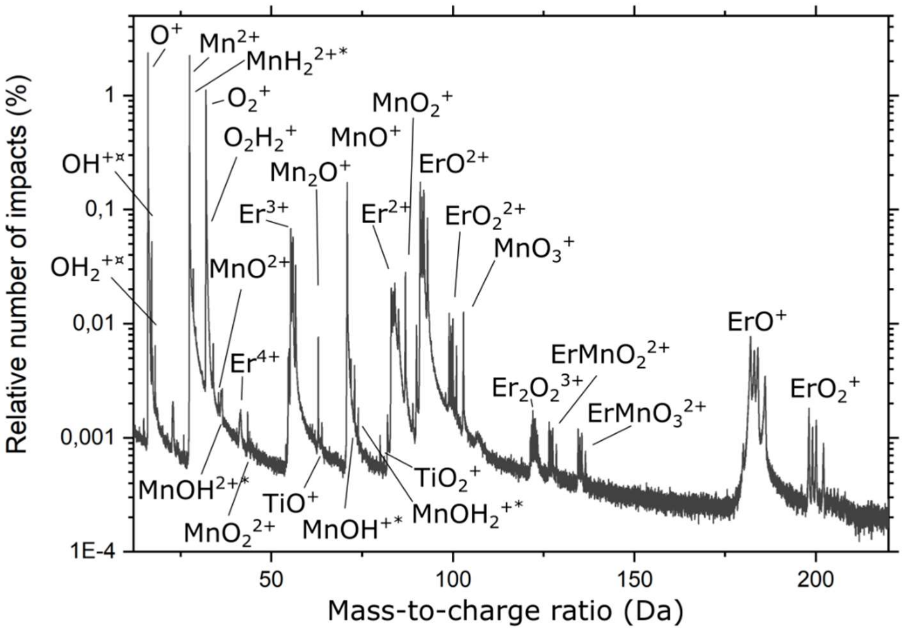

Supplementary Fig. 4 | Mass spectrum of Er(Mn,Ti)O_3 with labels for the ionic species. The horizontal axis shows the mass-to-charge ratio obtained from the APT time-of-flight measurements. The y-axis displays the relative number of ion impacts on the detectors in logarithmic scale. The asterisk "*" indicates H-species where the H-atom is not included in the reconstruction, i.e., MnOH is counted as MnO. ¤Denotes species that do not originate from the sample, but from the APT chamber. Species below 14 Da most likely originate from the APT chamber and are therefore not shown.

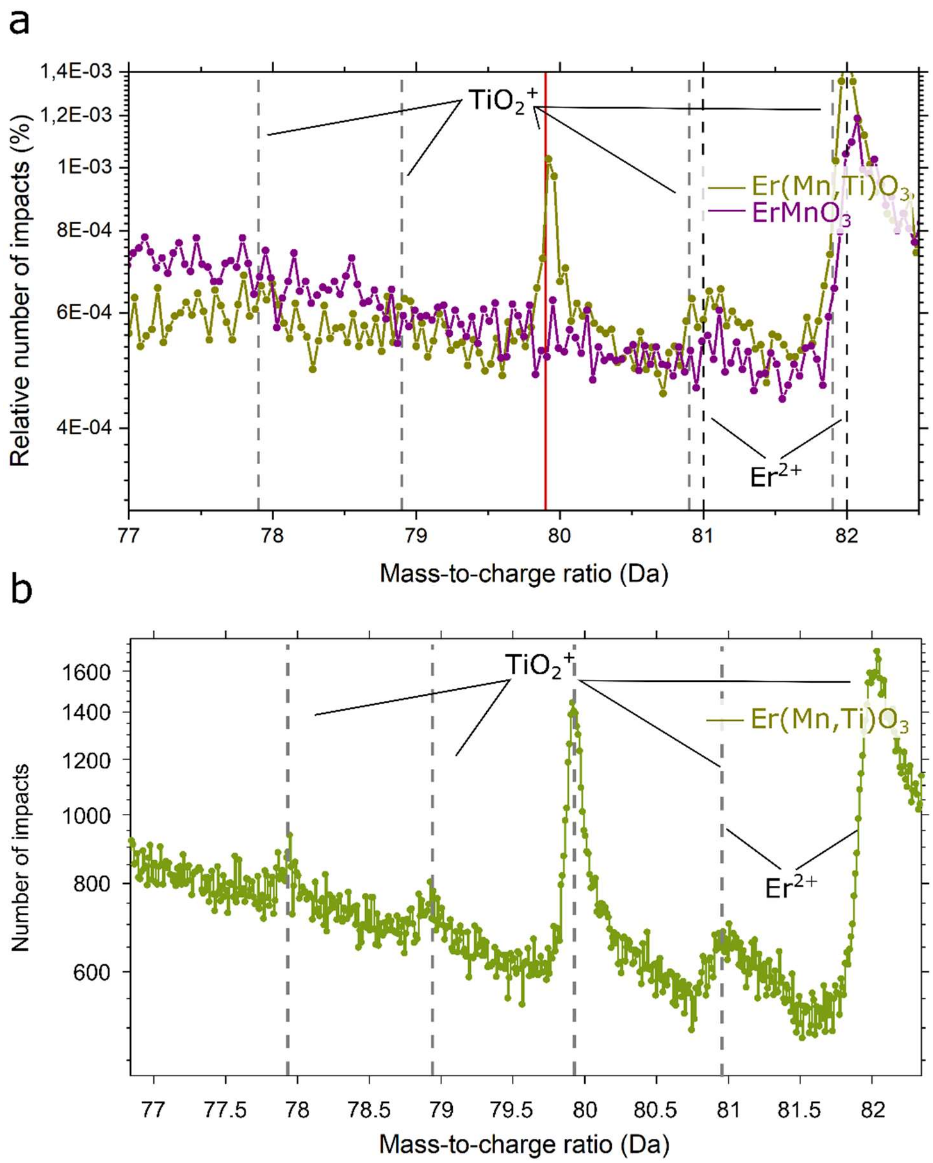

Supplementary Fig. 5 | Extended mass spectrum of TiO_2^+ ionic species. a, the graph represents an extension of Fig. 1e in the main text, where the visible peak of the ionic species of TiO_2^+ is shown, as well as the minor isotopes of TiO_2^+ (dashed gray lines). The latter are much less pronounced and partly overlapping with the Er^{2+} ionic species. **b**, a second dataset acquired using the same parameters where the minor isotopes are more pronounced proving that the main peak at 80 corresponds to TiO_2^+ .

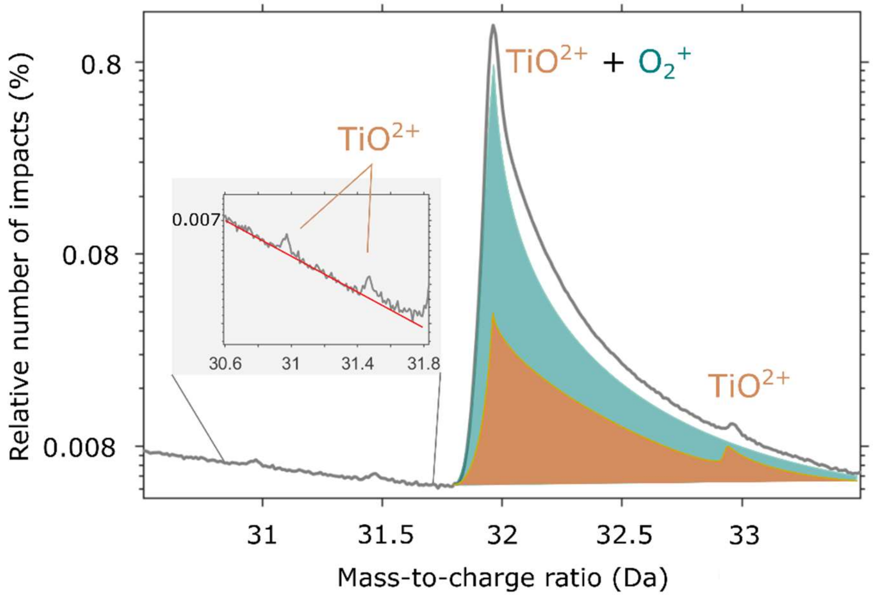

Supplementary Fig. 6 | Mass spectrum overlap of O_2^+ and TiO^{2+}. Mass spectrum centered around the 32 Da peak which contains both the O_2^+ and TiO^{2+} ionic species, as illustrated by the colored regions in the figure (orange is TiO^{2+} and blue is O_2^+). The O_2^+ is by far most dominant and hides the main TiO^{2+} peak, but the minor isotopes are visible. By using an exponential fit for the background (see inset, red line is illustrative), the total ion counts in the minor isotopes on the left can be used to deconvolute the main peak at 32 Da and extract an estimate for the TiO^{2+} concentration which is found to be 0.0138 at.%. Adding this to the previously measured Ti concentration of 0.0086 at.% the total concentration becomes 0.0224 at.%.

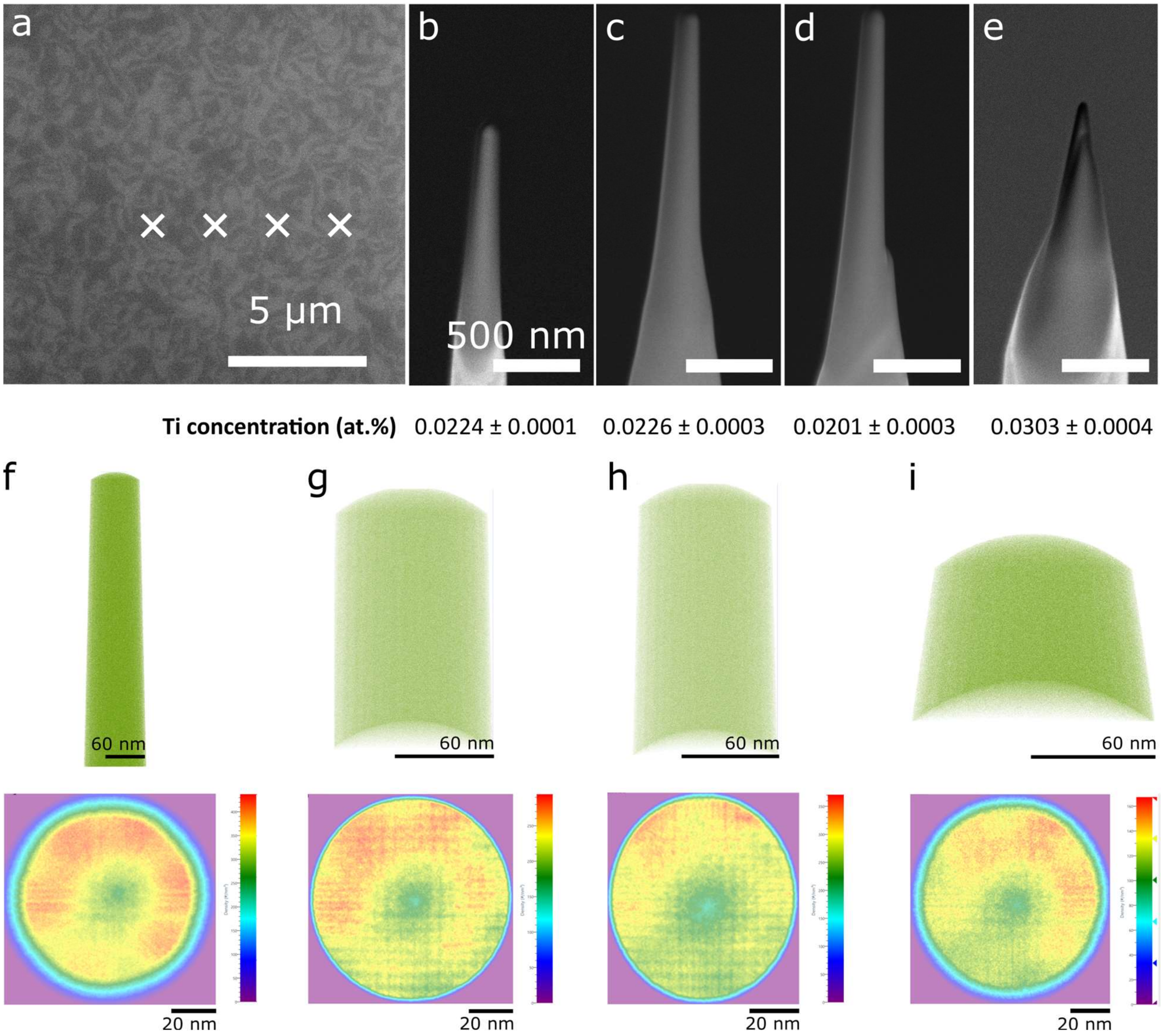

Supplementary Fig. 7 | Sample geometry and Ti concentration for different Er(Mn,Ti)O_3 needles. To evaluate spatial variations in Ti concentration, specimens were extracted from different locations within a region of about 20µm. a, SEM image of the bulk sample showing contrast from the ferroelectric domains (see Ref. [2]) and markers indicating typical distance between each of the needles in (b-e). Four specimens were analyzed with identical APT parameters of 5 pJ laser pulse energy, 250 kHz laser pulse frequency, 50 K temperature and 2% detection rate. Below the needles is the Ti concentrations and error estimates for each needle

(based on counting statistics) resulting in an average concentration of 0.0239 ± 0.0045 at.%. The value reflects only smaller variations in Ti concentration over micrometer distances, with an error bar that is the standard deviation based on the four values given and is likely to be dominated by the deviations in the shape of the needles, which determines the electric field strength at the apex. Figures (f-i) shows the atom maps with the corresponding field desorption map below from specimens in (b-e), in the same order.

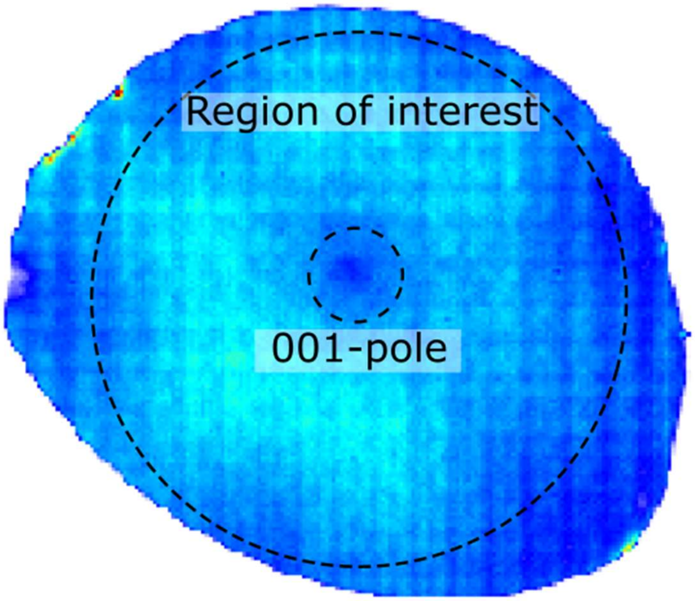

Supplementary Fig. 8 | Detector map. The image shows the detector view after completing the evaporation process. The map contains all the detected hits and is displayed as an image where bright (red) indicate more hits than dark (blue). The full region of interest is used for the reconstruction and for analyzing the full volume of the APT needle discussed in the main text, while the 001- pole is used for obtaining atomic resolution (roughly corresponding to the dotted lines). This pole corresponds to a facet on the surface of the needle formed during the field evaporation.

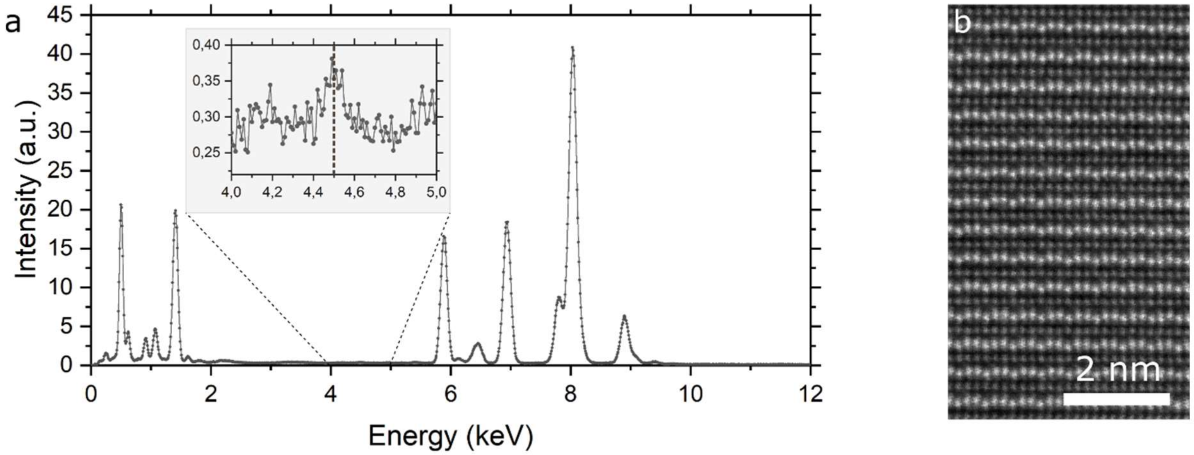

Supplementary Fig. 9 | EDX-TEM analysis of Er(Mn,Ti)O_3. a, The graph shows the complete EDX spectrum recorded on a Er(Mn,Ti)O_3 lamella (see main text and Methods for details). The inset shows the part of the spectrum with the Ti peak at 4.5 keV. The EDX spectrum represents the sum of $\approx$ 900 individual spectra recorded pixel-by-pixel along a single scan line with a length of 1.7 µm. b, HAADF-STEM image viewing along the a-axis gained from the same lamella as the EDX spectrum in a.

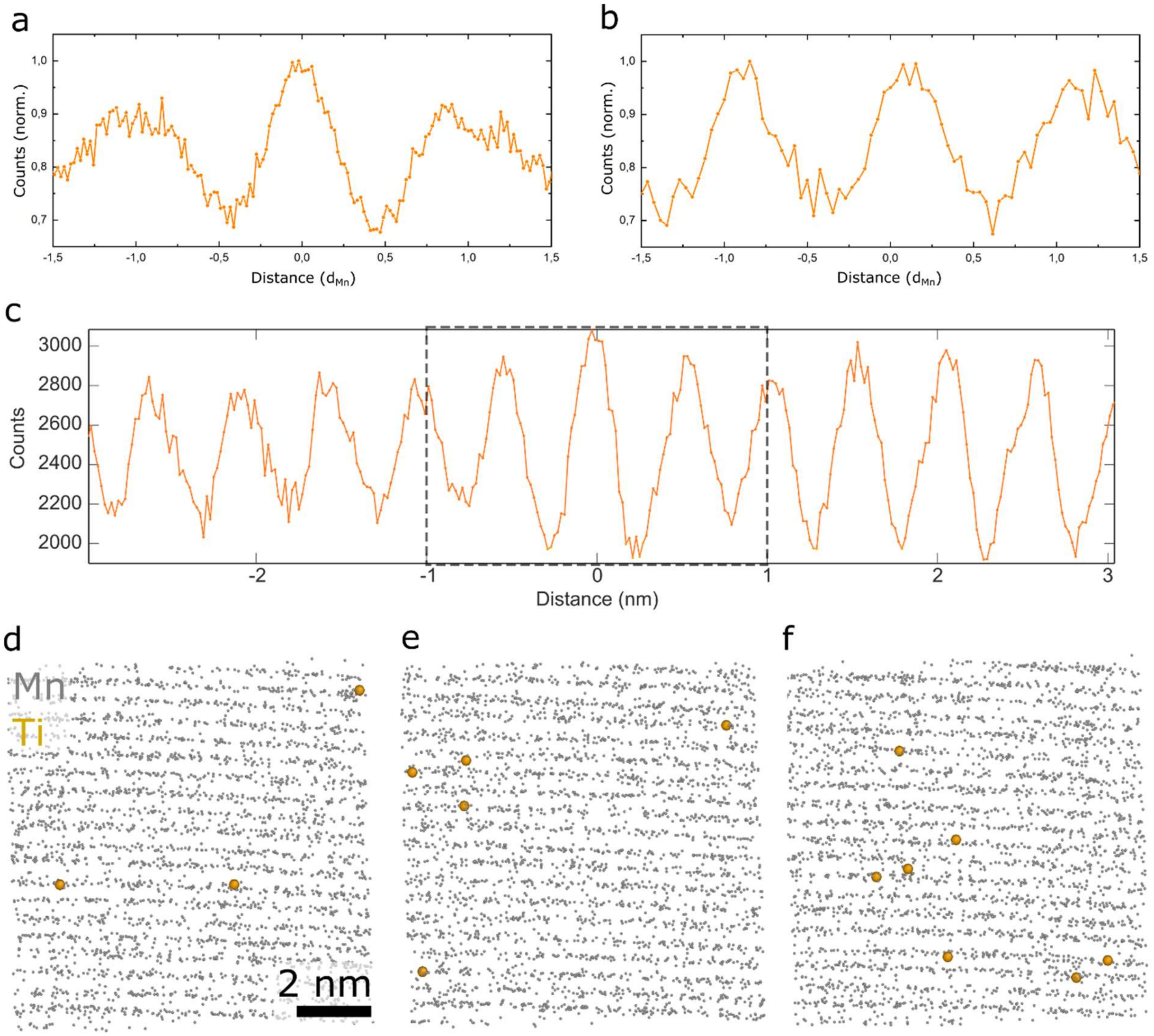

Supplementary Fig. 10 | Extended Ti-Mn SDM and 2D analysis of dopant position. a and **b,** TiO_2-Mn SDMs from two additional specimens extracted from different regions in of same bulk sample. The x-axis is given in terms of the Mn-Mn atomic plane distance. **c,** Extension of the SDM presented in Figure 3d, where 11 atomic planes are included. **d-f**, Selection of representative 2D projections from different areas, showing the distribution of Ti dopant atoms and their preference for occupying Mn lattice sites.

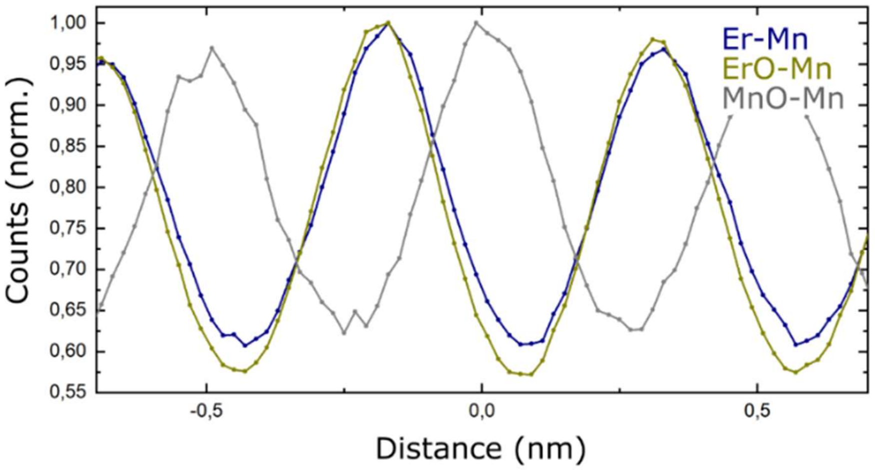

Supplementary Fig. 11 | Influence of O-molecular species in SDM analysis. Blue and green curves compare SDM data gained from molecular ErO and single Er ionic species, leading to qualitatively equivalent results. Similarly, the MnO-Mn SDM is calculated with the central peak around zero, indicating that MnO and Mn share the same atomic planes.

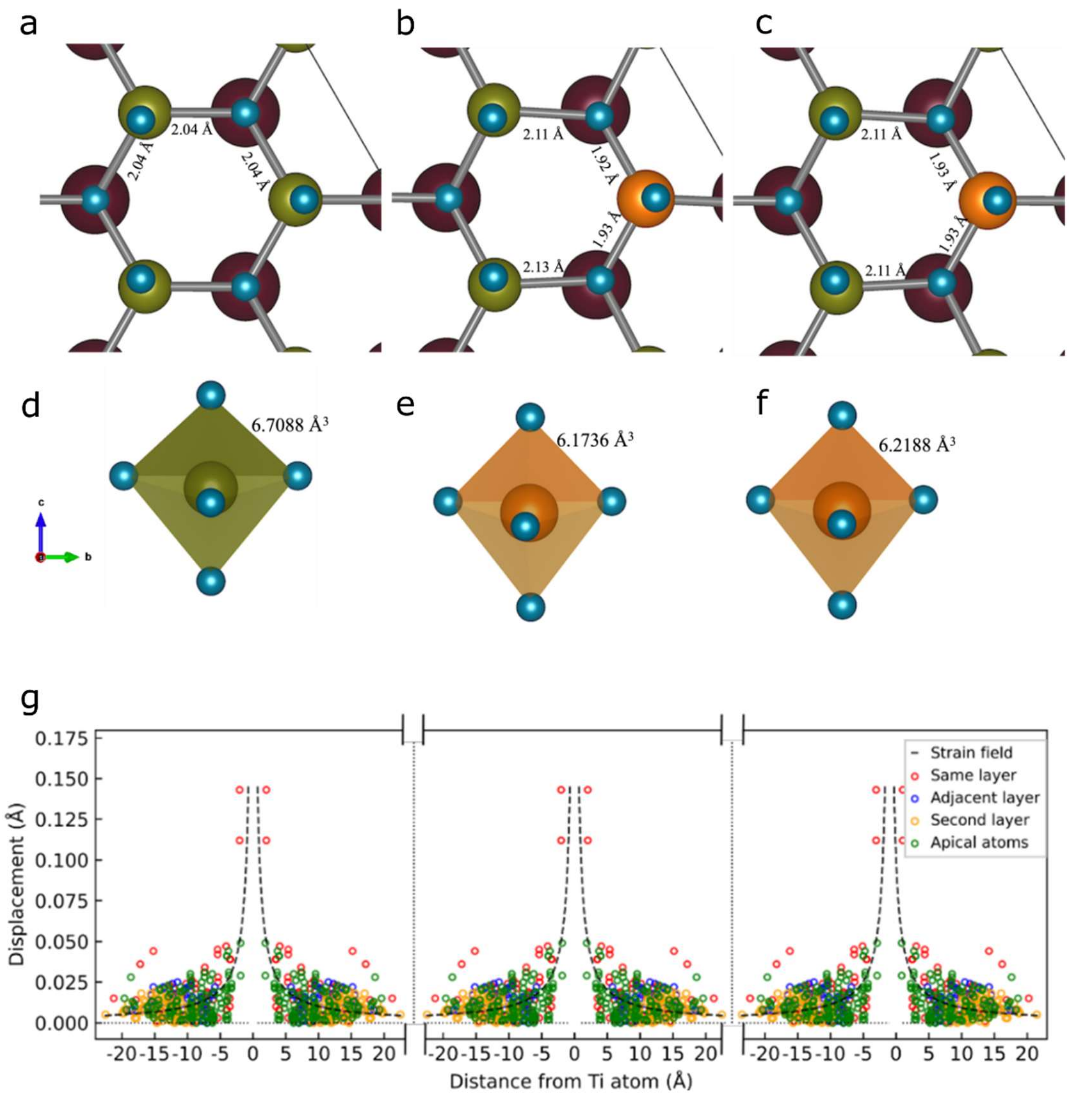

Supplementary Fig. 12 | Crystal perturbation upon Ti-doping. The resulting supercell of $ErMnO_3$ for a, stoichiometric undoped structure b, neutral supercell c, positively charged (+1) supercell and (d-f) shows the corresponding expansion and contraction of the $(Mn,Ti)O_3$ polyhedra at the doping site. g, Local strain field around Ti dopants visualized from DFT calculated ion displacements relative to the structure of undoped $ErMnO_3$ as a function of distance from a Ti dopant. Vertical dotted lines indicate boundaries between periodic supercells.

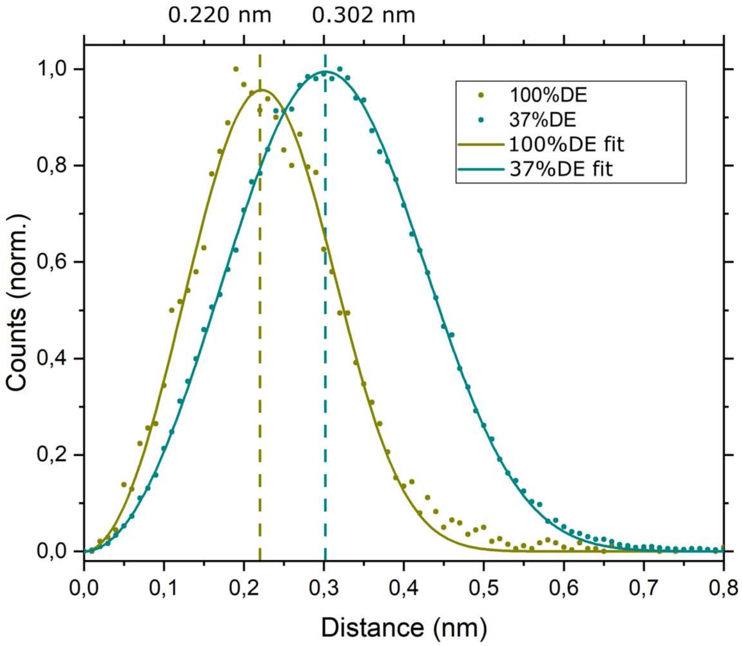

Supplementary Fig. 13 | Effect of detection efficiency on 1NN calculations. Calculated 1NN of Er from simulated datasets of undoped $ErMnO_3$ at two detection efficiencies (DE); 100% (blue) and 37% (yellow). Both datasets are fitted (solid line) and the simulation with low detection efficiency is closely matching the experimental data, meaning 37% DE is close to the real experimental conditions. The center of the fit can be seen to be shifted from 0.220 nm with 100% DE to 0.302 nm with 37% DE.

Supplementary References

1. Philippe, T. *et al.* Clustering and nearest neighbour distances in atom-probe tomography. *Ultramicroscopy* **109**, 1304–1309 (2009).
2. Hunnestad, K. A., Roede, E. D., Van Helvoort, A. T. J. & Meier, D. Characterization of ferroelectric domain walls by scanning electron microscopy. *J. Appl. Phys.* **128**, 191102 (2020).
