# Supplementary material for: Atomic-scale 3D imaging of individual dopant atoms in an oxide semiconductor
Source: Nat Commun. 2022 Aug 15;13:4783. doi: 10.1038/s41467-022-32189-0 (PMC9378652; doi:10.1038/s41467-022-32189-0)
Supplement: Supplementary file 3 — Lasing Reporting Summary [file 41467_2022_32189_MOESM3_ESM.pdf]

nature research

Corresponding author(s): Dennis Meier

# Lasing Reporting Summary

Nature Research wishes to improve the reproducibility of the work that we publish. This form is intended for publication with all accepted papers reporting claims of lasing and provides structure for consistency and transparency in reporting. Some list items might not apply to an individual manuscript, but all fields must be completed for clarity.

For further information on Nature Research policies, including our data availability policy, see Authors & Referees.

## ü Experimental design

**Please check: are the following details reported in the manuscript?**

1. Threshold

Plots of device output power versus pump power over a wide range of values indicating a clear threshold

- [ ] Yes
- [x] No

A commercial APT instrument (Cameca LEAP 5000XS) was used with built-in laser. All relevant parameters = settings are given in Methods so that the measurements can be reproduced.

2. Linewidth narrowing

Plots of spectral power density for the emission at pump powers below, around, and above the lasing threshold, indicating a clear linewidth narrowing at threshold

- [ ] Yes
- [x] No

Irrelevant. A commercial APT instrument (Cameca LEAP 5000XS) was used with built-in laser. All relevant parameters = settings are given in Methods so that the measurements can be reproduced.

Resolution of the spectrometer used to make spectral measurements

- [ ] Yes
- [x] No

Irrelevant. A commercial APT instrument (Cameca LEAP 5000XS) was used with built-in laser. All relevant parameters = settings are given in Methods so that the measurements can be reproduced.

3. Coherent emission

Measurements of the coherence and/or polarization of the emission

- [ ] Yes
- [x] No

Irrelevant. A commercial APT instrument (Cameca LEAP 5000XS) was used with built-in laser. All relevant parameters = settings are given in Methods so that the measurements can be reproduced.

4. Beam spatial profile

Image and/or measurement of the spatial shape and profile of the emission, showing a well-defined beam above threshold

- [ ] Yes
- [x] No

A commercial APT instrument (Cameca LEAP 5000XS) was used with built-in laser. All relevant parameters = settings are given in Methods so that the measurements can be reproduced.

5. Operating conditions

Description of the laser and pumping conditions
*Continuous-wave, pulsed, temperature of operation*

- [x] Yes
- [ ] No

Pulsed, f= 250 kHz, T = 25 - 50 K in APT chamber as described in Methods. A commercial APT instrument (Cameca LEAP 5000XS) was used with built-in laser. Additional info irrelevant. All relevant parameters = settings are given in Methods so that the measurements can be reproduced.

Threshold values provided as density values (e.g. W $cm^{-2}$ or J $cm^{-2}$) taking into account the area of the device

- [x] Yes
- [ ] No

2 and 30 pJ were used as described in Methods.

6. Alternative explanations

Reasoning as to why alternative explanations have been ruled out as responsible for the emission characteristics
*e.g. amplified spontaneous, directional scattering; modification of fluorescence spectrum by the cavity*

- [ ] Yes
- [x] No

Irrelevant. A commercial APT instrument (Cameca LEAP 5000XS) was used with built-in laser.

7. Theoretical analysis

Theoretical analysis that ensures that the experimental values measured are realistic and reasonable
*e.g. laser threshold, linewidth, cavity gain-loss, efficiency*

- [ ] Yes
- [x] No

Irrelevant. A commercial APT instrument (Cameca LEAP 5000XS) was used with built-in laser.

8. Statistics

Number of devices fabricated and tested

- [x] Yes
- [ ] No

Number of measured needles is explicitly given at the respective positions in the main text and supplementary.

Statistical analysis of the device performance and lifetime (time to failure)

☐ Yes
☒ No

Irrelevant for this study.
